# Dynamic Cerebral Autoregulation in Asymptomatic Patients With Unilateral Middle Cerebral Artery Stenosis

**DOI:** 10.1097/MD.0000000000002234

**Published:** 2015-12-31

**Authors:** Shuang Wang, Zhen-Ni Guo, Yingqi Xing, Hongyin Ma, Hang Jin, Jia Liu, Yi Yang

**Affiliations:** From the Stroke Center, Department of Neurology (SW, HM, HJ, YY); Neuroscience Center, Department of Neurology (Z-NG); Center for Neurovascular ultrasound (Y X), the First Hospital of Jilin Universit, Changchun, China and Shenzhen Institutes of Advanced Technology, Chinese Academy of Sciences, Xueyuan Avenue, Shenzhen University Town, Shenzhen, China (JL).

## Abstract

The aim of the study was to assess the capacity of dynamic cerebral autoregulation (dCA) in asymptomatic patients with unilateral middle cerebral artery (MCA) stenosis.

Fifty-seven patients with asymptomatic mild, moderate, and severe unilateral MCA stenosis and 8 patients with symptomatic severe unilateral MCA stenosis diagnosed by transcranial Doppler were enrolled. Twenty-four healthy volunteers served as controls. The noninvasive continuous cerebral blood flow velocity and arterial blood pressure were recorded simultaneously from each subject in the supine position. Transfer function analysis was applied to determine the autoregulatory parameters (phase difference [PD] and gain).

The PD values in the severe stenosis groups were significantly lower than those of the control group (60.71 ± 18.63°), the asymptomatic severe stenosis group was impaired ipsilaterally (28.94 ± 27.43°, *P* < 0.001), and the symptomatic severe stenosis group was impaired bilaterally (13.74 ± 19.21°, *P* < 0.001; 19.68 ± 14.50°, *P* = 0.006, respectively). The PD values in the mild and moderate stenosis groups were not significantly different than the controls (44.49 ± 27.93°; 48.65 ± 25.49°, respectively). The gain values in the mild and moderate groups were higher than in the controls (1.00 ± 0.58 cm/s/mm Hg vs 0.86 ± 0.34 cm/s/mm Hg, and 1.20 ± 0.59 cm/s/mm Hg vs 0.86 ± 0.34 cm/s/mm Hg, respectively). The gain values in the severe stenosis groups were significantly lower than that in the control group: the asymptomatic severe stenosis group was lower bilaterally (0.56 ± 0.32 cm/s/mm Hg, *P* = 0.003; 0.60 ± 0.32 cm/s/mm Hg, *P* < 0.05, respectively), whereas the symptomatic severe group was lower unilaterally (on the contralateral side) (0.53 ± 0.43 cm/s/mm Hg, *P* < 0.05).

In asymptomatic patients with unilateral MCA stenosis, only the dCA of the severe stenosis was ipsilaterally impaired. Acute stroke may aggravate the impaired dCA and even spread contralaterally.

## INTRODUCTION

Intracranial atherosclerosis is the most common vascular lesion in Asian, Hispanic, and African stroke patients.^1^ Due to the growing number of patients with intracranial atherosclerosis and the high risk of recurrence within 2 years after stroke,^2,3^ a better understanding of the changes of the pathology and pathogenesis of intracranial atherosclerosis is needed for targeted treatment and prevention.

Many researchers have focused on cerebral autoregulation in carotid cerebral stenosis. Impaired cerebral autoregulation was found in carotid cerebral stenosis, and the decrement of the autoregulation has a positive correlation with the degree of stenosis.^4–6^ Carotid endarterectomy, or stenting, has been proven to be effective in improving cerebral autoregulation in severe carotid cerebral stenosis.^7,8^ In contrast, relatively little is known about the cerebral autoregulation of the middle cerebral artery (MCA). Recently, Chen et al found that cerebrovascular reactivity and dynamic cerebral autoregulation (dCA) were impaired in MCA stenosis through transfer function analysis.^9^ However, there are few studies regarding dCA in asymptomatic patients with different degrees of unilateral MCA stenosis, the relationship between the relevant and changing hemodynamic parameters and the risk of MCA territory ischemic stroke. Additionally, different arterial remodeling modes on high-resolution magnetic resonance imaging were observed between symptomatic and asymptomatic MCA stenosis.^10,11^ The changes in the vascular structures may influence the cerebral hemodynamics. However, it is unknown whether these changes can affect dCA.^12^

In this study, we attempt to assess the dCA in asymptomatic patients with different degrees of unilateral MCA stenosis by the autoregulation parameters using transfer function analysis.

## METHODS

### Participants

The informed consent was obtained from all participants and the Ethics Committee of the First Hospital of Jilin University approved the study design.

We conducted a prospective study of consecutive patients with unilateral stenosis in the M1 segment of the MCA diagnosed by transcranial Doppler. The patients were recruited from September 2013 to December 2013 at the First Norman Bethune Hospital of Jilin University. Patients were included in this study if they (1) had unilateral MCA stenosis; (2) had a sufficient bilateral temporal bone window for the insonation of the MCA; and (3) were 18 to 80 years old. Patients were excluded from this study if they: (1) had a history of transient ischemia attack or stroke; (2) had other intracranial or/and extracranial major vascular stenosis/occlusion; (3) had a history of atrial fibrillation, myocardial infarction, unstable angina, and valvular heart disease; or (4) had a history of diabetes mellitus, impaired renal function, anxiety disorder, migraine, or peripheral neuropathy. Twenty-eight healthy volunteers (age- and sex-matched) were included as control subjects.

Extracranial and intracranial artery stenosis or occlusion was diagnosed by transcranial Doppler and magnetic resonance angiography. The degree of stenosis was classified according to the scheme of Chen et al.^13^ Peak systolic velocity of >160 to 200 cm/s indicated mild stenosis (group 1; n = 22; men, 12); peak systolic velocity of >200 to 280 cm/s indicated moderate stenosis (group 2; n = 13; men, 8); and peak systolic velocity of >280 cm/s indicated severe stenosis (group 3; n = 30; men, 25). The results of magnetic resonance angiography were used as a reference. Each symptomatic patient was diagnosed with acute stroke according to clinical symptoms and the magnetic resonance imaging, magnetic resonance angiography, and transcranial Doppler results. The dCA was performed 5 to 10 days after onset. Each patient was diagnosed with MCA stenosis by 2 neurologists who were blinded to this study.

### Study Protocol

During the study, all of the subjects were in the supine position with their heads slightly elevated and were kept quiet for 10 min. Then, we measured the baseline blood pressure at the brachial artery (automatic blood pressure monitor, Omron 711). The cerebral blood flow velocity of bilateral MCAs by a 2-MHz probe headframe of transcranial Doppler (MultiDop X2, DWL, Sipplingen, Germany) was continuously recorded at a depth of 45 to 55 mm distal to the M1 stenosis, and the arterial blood pressure was simultaneously recorded using servo-controlled plethysmograph (Finometer Pro, Netherlands) on the middle finger for 10 min. End-tidal CO_2_ was monitored using a capnograph (MultiDop X2, DWL, Sipplingen, Germany) with a face mask attached to the nasal cannula. The recorded data were then used to assess the dCA.

All subjects were asked to avoided smoking, drinking, and caffeine for at least 12 h before the examination. The examination was performed in a quiet, dedicated research laboratory at a controlled temperature of 20 to 24°C, with minimal external stimuli.

### Data Analysis

All data were stored and processed using MATLAB (Version R2009b, MathWorks, Inc, United States). The raw data were recorded in the binary format with sampling frequency of 1000 Hz for CBFV and 100 Hz for arterial blood pressure, respectively. CBFV recordings were first decimated to the same sampling frequency of ABP at 100 Hz. They were then synchronized with arterial blood pressure by shifting the time lags derived from a cross-correlation function. The recordings were further down-sampled to 1 Hz after filtered by a 3rd order Butterworth low-pass filter (cutoff at 0.5 Hz). The transfer function can then be estimated by dividing the cross-spectrum of the 2 signals by the autospectrum of arterial blood pressure in frequency domain. The phase difference (PD) and gain can then be derived from the real and imaginary parts of the transfer function. We also estimated the coherence function between the 2 signals and only considered the cases for further statistical analyses if the averaged coherence is > 0.4 within the range of 0.06 to 0.12 Hz.^14^

### Statistical Analysis

The Shapiro–Wilk test was used to determine the distribution of the data, and all the variables had a normal distribution. Statistical descriptions of all the variables are presented as the mean ± SD. Analysis of variance and the Dunnett *t* test were performed to compare the continuous variables across groups and for multiple comparison, and Student's *t* test was used for comparisons at the individual level. The chi-square test was used to identify count data. Any *P* value <0.05 was considered statistically significant. All statistical analyses were performed with SPSS 18.0 (IBM, West Grove, PA).

## RESULTS

### Demographic Information

In total, 65 patients with unilateral MCA stenosis were enrolled in this study. According to the peak systolic velocity detected by transcranial Doppler, the patients were divided into 3 groups: mild stenosis (49.59 ± 7.33 years; 12 men and 10 women; 10 patients with left MCA stenosis and 12 patients with right MCA stenosis), moderate stenosis (50.92 ± 10.28 years; 7 men and 6 women; 8 patients with left MCA stenosis and 5 patients with right MCA stenosis), and severe stenosis (asymptomatic: 48.27 ± 5.43 years, 18 men and 4 women, 12 patients with left MCA stenosis and 10 patients with right MCA stenosis; symptomaitc: 49.34 ± 4.32 years, 5 men and 3 women, 5 patients with left MCA stenosis and 3 patients with right MCA stenosis). The symptomatic patients were acute MCA territory infarctions, and the dCA was performed 5 to 10 days after onset. Twenty-four mentally and physically healthy volunteers (48.34 ± 7.2 years; 12 males) served as controls. Using the chi-square test, we found significant differences in the drinking history (*χ*^2^ = 12.780, *P* = 0.012) and smoking history (*χ*^2^ = 14.255, *P* = 0.007) of the groups. There were no significant differences in age, sex, hypertension, and end-tidal CO_2_ among the groups. The baseline characteristics are presented in Table 1. The mean follow-up was 288 days. Neither ischemic stroke nor transient ischemia attack occurred.

**TABLE 1:**
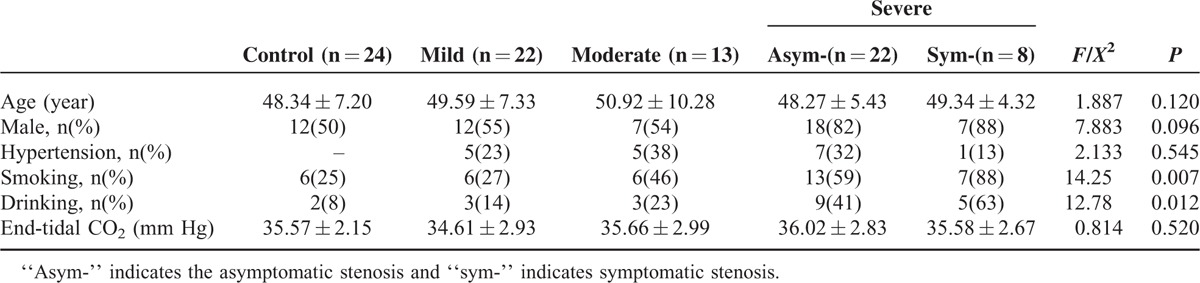
Baseline Characteristics

### Dynamic Cerebral Autoregulation

#### Phase Difference

**FIGURE 1:**
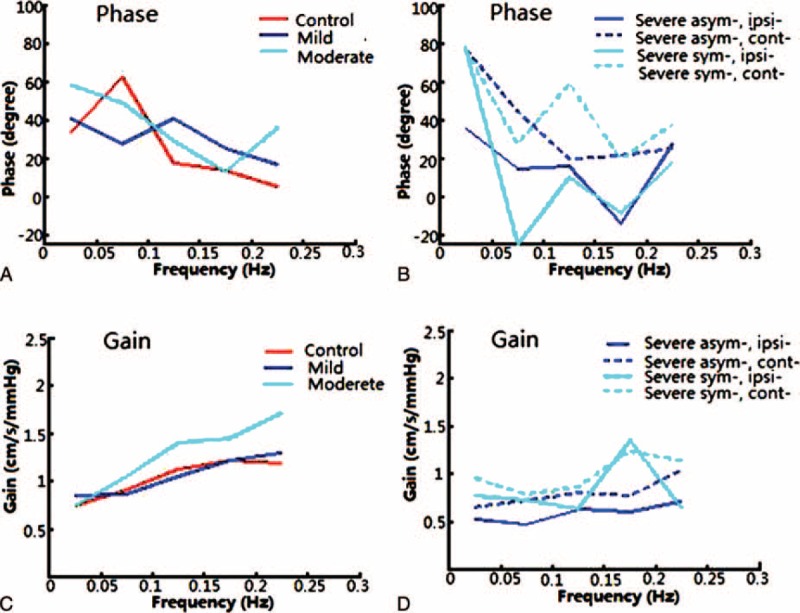
The autoregulatory parameters, phase difference, and gain derived from the transfer function and estimated over a range (0.06–0.12 Hz). Each colored line denotes an average parameter for each group. (A) There was no significant difference in the phase difference between the control group, the mild stenosis group, and the moderate stenosis group. (B) The phase difference in the asymptomatic severe stenosis group was reduced ipsilaterally and was reduced bilaterally in the symptomatic severe stenosis group. (C, D) The gain in the asymptomatic severe stenosis group was reduced bilaterally, whereas in the symptomatic severe group was lower unilaterally (on the contralateral side). “Asym-” indicates the asymptomatic stenosis and “sym-” indicates symptomatic stenosis. “Ipsi-” indicates the ipsilateral side and “cont-” indicates contralateral side.

**FIGURE 2:**
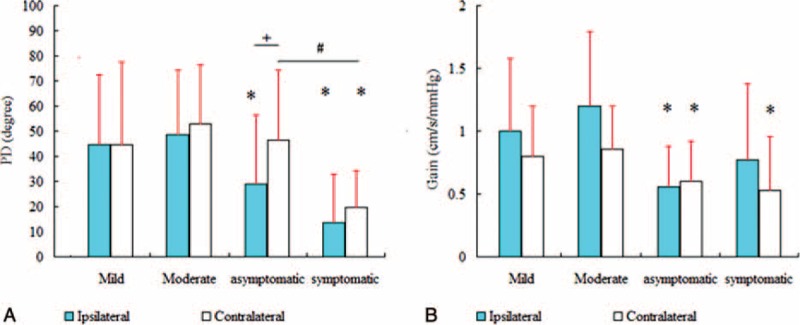
Statistical distributions of the autoregulatory parameters for each category. (A) The phase difference of the asymptomatic severe stenosis group was significantly reduced ipsilaterally, and the phase difference of the symptomatic severe stenosis group was significantly reduced bilaterally compared to the control group. The phase difference from the ipsilateral side was significantly lower than from the contralateral side in the asymptomatic severe stenosis group. The phase difference from the contralateral side of the symptomatic severe stenosis group was significantly lower than the phase difference from the contralateral side of the asymptomatic severe stenosis group. (B) The gain in the asymptomatic severe stenosis group was lower bilaterally, whereas in the symptomatic severe group was lower unilaterally (on the contralateral side). Asterisk (∗) indicates a significant difference of each group when comparing with the control group. Hash (#) indicates a significant difference between the asymptomatic and the symptomatic groups. Plus (+) indicates a significant difference between the ipsilateral and the contralateral sides. PD = phase difference.

There was no significant difference in the PD between the ipsilateral and contralateral side in the mild stenosis group (44.49 ± 27.93° vs 44.79 ± 32.88°, *P* = 0.954), the moderate stenosis group (48.65 ± 25.49° vs 52.81 ± 23.71°, *P* = 0.462), and the symptomatic severe stenosis group (13.74 ± 19.21° vs 19.68 ± 14.50°, *P* = 0.518, Table 2, Figure 1A, B and Figure 2A). However, in the asymptomatic severe stenosis group, the PD on the ipsilateral side was 28.94 ± 27.43°, which was significantly lower than the contralateral side (46.32 ± 28.04°, *P* = 0.021, Table 2, Figure 1B and Figure 2A).

**TABLE 2:**
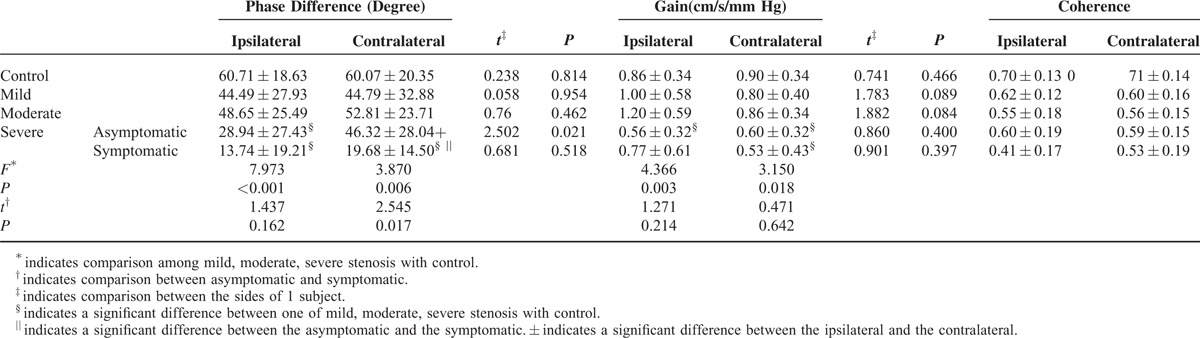
Phase Difference, Gain, and Coherence in Controls and Different Degree Middle Cerebral Artery Stenosis

There was a trend, although not significant, toward lower PD values in the symptomatic severe stenosis group than the asymptomatic severe stenosis group (13.74 ± 19.21° vs 28.94 ± 27.43°, *P* = 0.162, Table 2) on the ipsilateral side. In contrast, on the contralateral side, the symptomatic severe stenosis group had significantly lower values than the asymptomatic severe stenosis group (19.68 ± 14.50° vs 46.32 ± 28.04°, *P* = 0.017, Table 2, Figure 1 and Figure 2A).

There was a significant difference in the PD between the severe stenosis group and the control group (*P* < 0.001). The asymptomatic stenosis group was impaired on the damaged side, and the symptomatic stenosis group was impaired on both the damaged and contralateral sides (*P* < 0.001, *P* < 0.05, respectively, Table 2, Figure 1B and Figure 2A).

#### Gain

The gain estimated from the controls and the patients were all in the shape of a high-pass filter, suggesting that autoregulation was active. The gains in the mild and moderate groups were higher than in the controls (1.00 ± 0.58 cm/s/mm Hg vs 0.86 ± 0.34 cm/s/mm Hg, and 1.20 ± 0.59 cm/s/mm Hg vs 0.86 ± 0.34 cm/s/mm Hg, respectively, Table 2, Figure 1C and Figure 2B). The gain in the severe stenosis group was significantly lower than in the control group: the asymptomatic severe stenosis group was lower bilaterally (0.56 ± 0.32 cm/s/mm Hg, *P* = 0.003; 0.60 ± 0.32 cm/s/mm Hg, *P* < 0.05, respectively), whereas the symptomatic severe group was lower unilaterally (on the contralateral side, 0.53 ± 0.43 cm/s/mm Hg, *P* < 0.05, Table 2, Figure 1D and Figure 2B).

#### Coherence

There were no significant differences between the cases and controls in terms of coherence (Table 2).

## DISCUSSION

The main findings of this study are that in unilateral MCA stenosis: (1) only in the severe MCA stenosis groups, the PD values were significantly lower compared with the control group. However, the asymptomatic stenosis group was impaired on the damaged side, and the symptomatic stenosis group was impaired on both the damaged and contralateral sides. (2) On the damaged side, there was a trend, although not significant, toward lower PD values in the symptomatic severe stenosis group than in the asymptomatic severe stenosis group, but on the contralateral side, the symptomatic severe stenosis group had significantly lower PD values than the asymptomatic severe stenosis group.

The PD represents the response of blood flow to the change of blood pressure. Generally, a large positive PD of 30 to 70° can be regarded as intact autoregulation, whereas a PD of 0° can be considered to be completely impaired autoregulation.^15^ In the present study, we found that in the severe asymptomatic MCA stenosis group, the PD was significantly lower than in the healthy group, which is consistent with the findings of previous studies. Haubrich et al showed that the M-wave PD was reduced with increasing degrees of M1 stenosis of the MCA by cross-spectral analysis.^16^ Using the thigh stuff technique and the autoregulation index, Gong et al found that cerebral autoregulation was impaired in patients with severe stenosis in the MCA and insufficient collateral compensation.^17^ Recently, Chen et al revealed that the cerebrovascular reactivity and dCA were impaired in MCA stenosis using transfer function analysis.^9^

In addition, compared with the asymptomatic severe stenosis group, the PD of the symptomatic severe MCA stenosis group had a trend, although not significant, toward poorer values. Gong et al also suggested that the autoregulation index was significantly reduced in severe stroke patients compared with asymptomatic or transient ischemia attack patients.^17^ The dCA was significantly lower on the affected side than the contralateral side in asymptomatic patients, which is consistent with the findings of previous studies.^7,9,18^ The dCA was impaired bilaterally in symptomatic patients with MCA territory infarcts. Other studies have found that the dCA tended to be impaired bilaterally and remained impaired for at least 1 to 2 weeks.^19,20^ Additionally, some studies have found that the impairment of dCA may spread to the contralateral side mainly in 5 to 14 days following onset.^14,21,22^ With the differences of dCA between the symptomatic and asymptomatic severe stenosis groups, we believe that acute ischemic stroke can aggravate the impaired dCA.

The gain has been regarded to be an indicator of the change of amplitude in cerebral blood flow velocity corresponding to the changes in arterial blood pressure. In general, increasing the gain should indicate less filtering with more transmission of amplitude changes and thus poorer autoregulation, whereas a lower gain is considered to represent improved autoregulation. However, in our study, it was worth noting that the gain increased for the mild and moderate MCA stenosis groups and decreased for the severe MCA stenosis group compared with the controls, and this finding was similar with that of a previous study.^9^ Several factors could lead to the lower gain in patients with MCA stenosis. First, due to atherosclerosis, the patients may have higher cerebral vascular resistance, which could enhance the effective damping of rises in blood pressure. Thus, it could lead to lower gain values. Second, due to the effects of stenosis, there is post-stenotic vasodilatation, and these changes may result in lower vasodilatory. Therefore, the lower gain in the MCA stenosis did not mean that there was an improved dCA. Therefore, it is important to consider changes in PD and gain together when using transfer function analysis to measure the dCA.

Another striking feature of this study was the low incidence of stroke and transient ischemia attack, particularly in patients with a severely impaired dCA. In our follow-up study, no patient experienced stroke or transient ischemia attack. Similar results were reported by Ni^23^ and Kremer.^24^ They observed that asymptomatic MCA stenosis appeared to be a benign prognosis with a low risk of ipsilateral stroke. They attributed the mechanism of the low stroke risk to the stability of the MCA plaque, which has been confirmed by many studies of the transcranial Doppler using microembolic detection. These studies found that there were no or few microembolic signals detected in asymptomatic MCA stenosis compared with symptomatic MCA stenosis.^23,25,26^ Both artery-to-artery embolism and hypo-perfusion with impaired embolism clearance play important roles in intracranial atherosclerotic stroke.^27^ An intact dCA can maintain stable cerebral perfusion and embolism clearance functioning.^28^ In our study, we found that the dCA of the asymptomatic stenosis group was better than that of the symptomatic stenosis group, although the finding was not significant due to the small number of patients. The relatively preserved dCA may contribute to the low incidence of stroke in asymptomatic MCA stenosis patients compared to symptomatic patients.

A major limitation of the present study is the small number of patients examined. The other limitation is the follow-up time is not long enough. Further long-term follow-up research should be performed to link the dCA to the risk of artery atherosclerotic stenosis and treatment (medical and stenting).

## CONCLUSION

In a comparison of the dCA between the asymptomatic and symptomatic severe MCA stenosis groups, we inferred that ischemic stroke could aggravate dCA impairment. There is clearly a need for prospective, multicenter, large-scale trials of the dCA in atherosclerotic stenosis and stroke patients.
